# Supplementary material for: Decrease in Soil Functionalities and Herbs’ Diversity, but Not That of Arbuscular Mycorrhizal Fungi, Linked to Short Fire Interval in Semi-Arid Oak Forest Ecosystem, West Iran
Source: Plants (Basel). 2023 Mar 1;12(5):1112. doi: 10.3390/plants12051112 (PMC10005139; doi:10.3390/plants12051112)
Supplement: Supplementary file 1 [file plants-12-01112-s001.zip › plants-2226202-supplementary.pdf]

Supplementary Information

Decrease in soil functionalities and herbs' diversity, but not that of arbuscular mycorrhizal fungi, linked to short fire interval in semi-arid oak forest ecosystem, west Iran

Javad Mirzaei[a*], Mehdi Heydari[a], Reza Omidipour[b],

Nahid Jafarian[a], Christopher Carcaillet[cde]

[a] Department of Forest Science, Faculty of Agriculture, Ilam University, Ilam, Iran

[b] Department of Rangeland and Watershed Management, Faculties of Natural Resources and Earth Sciences, Shahrekord University, 8818634141, Shahrekord, Iran

[c] Ecole Pratique des Hautes Etudes (EPHE), Paris Sciences & Lettres Université (PSL), F-75014 Paris, France

[d] Univ Lyon, Université Claude Bernard Lyon 1, CNRS, ENTPE (UMR 5023 LEHNA), F-69622 Villeurbanne, France

[e] Department of Plant Sciences, University of Cambridge, Cambridge, UK

* Author for correspondences

Table S1. The eigenvalue, percent of explained variance and cumulative variance for different axes (or components) of the PCA analysis.

PCA axis	Eigenvalue	Variance (%)	Cumulative variance (%)
1	5.945	45.73	45.73
2	2.470	19.00	64.73
3	1.485	11.42	76.15
4	1.082	8.32	84.47
5	0.702	5.40	89.88
6	0.495	3.81	93.69
7	0.317	2.44	96.13
8	0.202	1.55	97.68
9	0.172	1.32	99.00
10	0.082	0.63	99.63
11	0.029	0.22	99.86
12	0.013	0.10	99.96
13	0.005	0.04	100.00

Table S2. Correlation coefficients and p-values between first two axes of PCA and different soil physical, chemical and biological properties, and diversity indices of herbaceous and fungal taxa. BD: bulk density; EC: electrical conductivity; pH: soil acidity; N: total nitrogen; MBC: microbial biomass carbon; SR: soil basal respiration; SIR: substrate-induced respiration; SOM: soil organic matter, R_Plant: plant richness, H_Plant: Shannon index of plants, D_Plant: Gini-Simpson index of plants, H_AMF: Shannon index of fungi.

Axis 1			Axis 2		
Variables	*r*	*p*-value	Variables	*r*	*p*-value
SR	0.94	< 0.001	H_Plant	0.80	< 0.001
N	0.93	< 0.001	D_Plant	0.67	0.001
Urease	0.91	< 0.001	R_Plant	0.66	0.001
SIR	0.89	< 0.001	BD	0.50	0.023
MBC	0.88	< 0.001	EC	-0.66	0.001
SOM	0.59	< 0.001			
D_Plant	0.56	0.011			
pH	-0.48	0.033			
H_AMF	-0.49	0.028			
BD	-0.65	0.002			

Table S3. Direct and indirect standardized effects of fire number on different variables of soil physical, chemical and biological properties, and diversity indices of herbaceous and fungal taxa. Significant paths ($p < 0.05$) are indicated in bold.

Response	Indicator	Mediator	Pathway	Predictor	*p*	Path coefficient
Physical properties	BD	-----	Direct	Fire no	**<0.001**	**0.85**
Chemical properties	EC	-----	Direct	Fire no	**0.026**	**-0.46**
Biological properties	SIR	-----	Direct	Fire no	**<0.001**	**-0.68**
Plant diversity	Richness	-----	Direct	Fire no	**0.05**	**-0.38**
Fungal diversity	Shannon I.	-----	Direct	Fire no	**0.043**	**0.39**
Fungal diversity	Shannon I.	-----	Direct	Chemical properties	**0.021**	**-0.41**
Fungal diversity	Shannon I.	Chemical properties	Indirect	Fire no	**0.05**	**0.194**

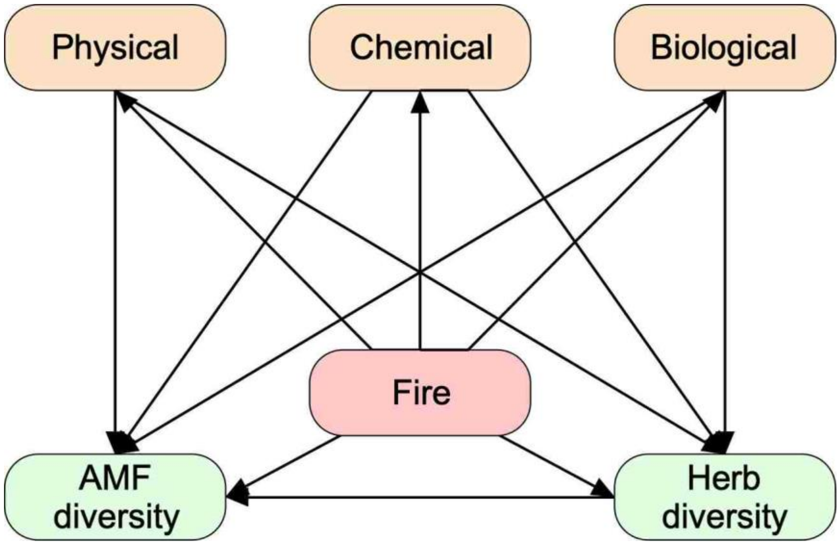

Figure S1. The schematic diagram of SEM model for direct and indirect effects of fire treatment of different frequencies on the best variables in different groups of soil physical, chemical and biological and plant and fungal species diversity indices.

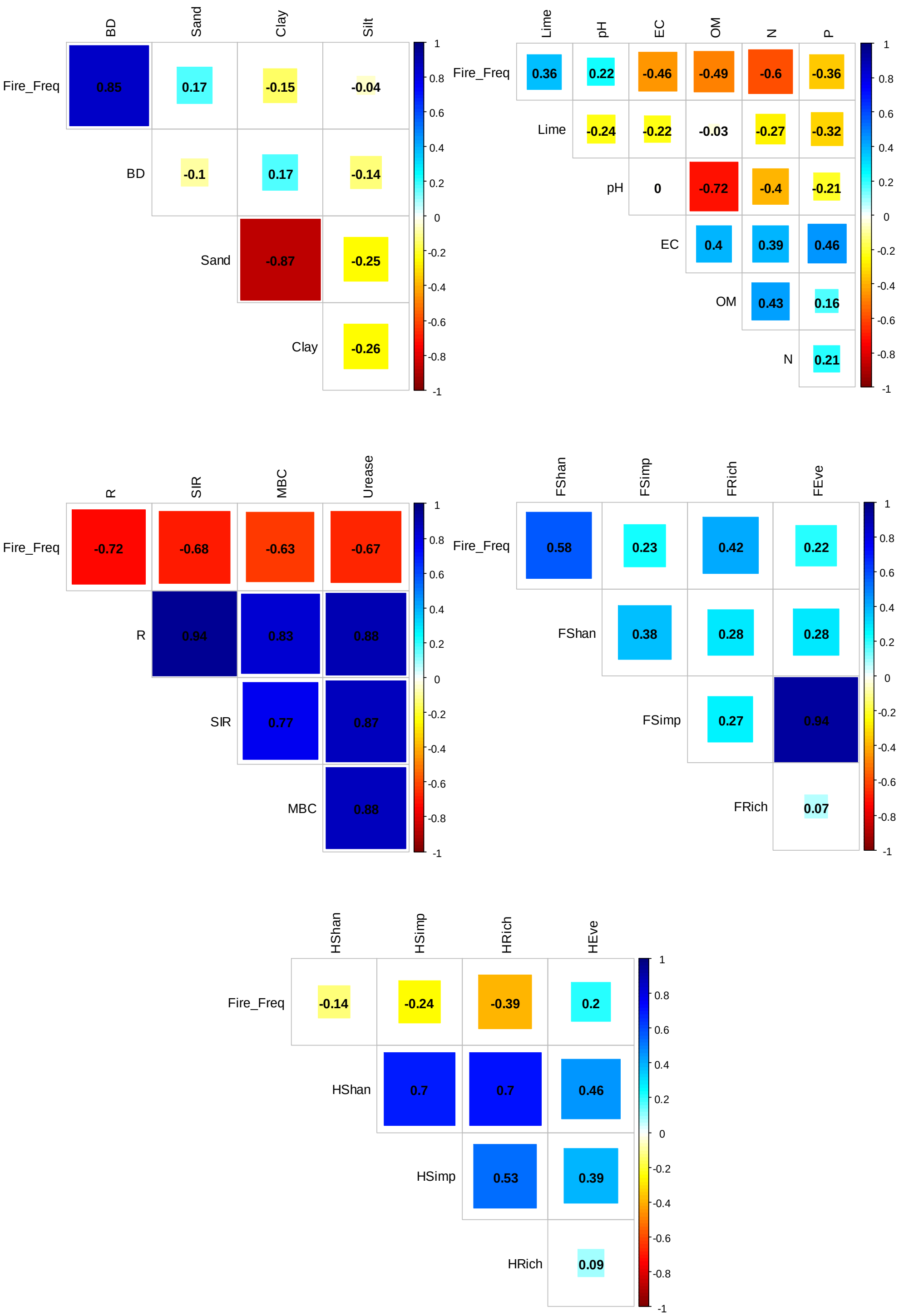

Figure S2. Spearman correlation coefficients between fire number and the different variables of soil physical, chemical and biological, and diversity indices of herbaceous and fungal taxa for per-selection of the best variables in SEM model. Blue to red color indicates negative to positive correlation, the numbers within squares represent correlation coefficient.
